# Sub-10-fs population inversion in N_2_^+^ in air lasing through multiple state coupling

**DOI:** 10.1038/ncomms9347

**Published:** 2015-09-25

**Authors:** Huailiang Xu, Erik Lötstedt, Atsushi Iwasaki, Kaoru Yamanouchi

**Affiliations:** 1 Department of Chemistry, School of Science, The University of Tokyo, 7-3-1 Hongo, Bunkyo-ku, Tokyo 113-0033, Japan; 2 State Key Laboratory on Integrated Optoelectronics, College of Electronic Science and Engineering, Jilin University, Changchun 130012, China

## Abstract

Laser filamentation generated when intense laser pulses propagate in air has been an attractive phenomenon having a variety of potential applications such as detection and spectroscopy of gases at far distant places. It was discovered recently that the filamentation in air induces ‘lasing', showing that electronically excited N_2_^+^ is population-inverted, exhibiting marked contrast to the common understanding that molecular ions generated by intense laser fields are prepared mostly in their electronic ground states. Here, to clarify the mechanism of the population inversion, we adopt few-cycle laser pulses, and experimentally demonstrate that the lasing at 391 nm occurs instantaneously after N_2_^+^ is produced. Numerical simulations clarify that the population inversion is realized by the post-ionization couplings among the lowest three electronic states of N_2_^+^. Our results shed light on the controversy over the mechanism of the air lasing, and show that this post-ionization coupling can be a general mechanism of the atmospheric lasing.

## Abstract (web summary)

Remote generation of population-inverted gain media in air is a step towards the realization of bright and coherent atmospheric lasers. Here, the authors verify population inversion in N_2_^+^ and demonstrate the generation of air lasing by acting on it as the gain medium.

## Graphical abstract

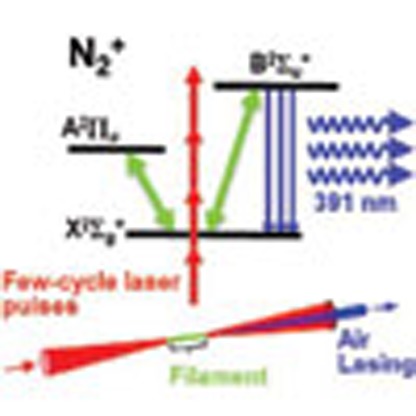

Remote generation of population-inverted gain media in air is a promising step towards the realization of bright and coherent atmospheric lasers for various applications such as standoff spectroscopy^1^, sensing^2^,^3^,^4^ and remote material processing^5^. Several approaches have been developed so far to remotely build up the population inversion of atmospheric constituents, based on which lasing actions in air, popularly referred to as ‘air lasing', have been achieved in both backward and forward propagation direction^2^,^3^,^4^,^6^,^7^,^8^,^9^,^10^. In the early demonstrations of air lasing actions, both the major atmospheric constituents, that is, molecular oxygen and nitrogen, were employed as the gain media. With the pump of deep-ultraviolet 100 ps pulses at 226 nm, molecular oxygen in air undergoes two-photon dissociation. Subsequent excitation of atomic oxygen fragments via a resonant two-photon process results in the population inversion between the 3*p* and 3*s* levels, giving rise to high-gain amplified spontaneous emission at 845 nm^3^. For neutral nitrogen molecules, both amplified spontaneous emission and seed amplification of the C^3^Π_u_(*ν*=0)→B^3^Π_g_(*ν*=0) transition at 337 nm were realized in laser filaments as the result of the population inversion achieved by the excitation schemes of the optically driven electron impact by femtosecond circularly or picosecond linearly polarized pulses^8^,^9^,^10^.

Recently, it was reported that coherent narrow-band B^2^Σ_u_^+^–X^2^Σ_g_^+^ (*v*′, *v*′′) emissions of nitrogen molecular ions N_2_^+^ were also observed when pure nitrogen or air was irradiated with intense laser pulses^6^,^11^,^12^,^13^. These coherent emissions were found to propagate in the forward direction along the pump laser beam and exhibited excellent temporal and spatial coherence properties. It was further revealed that the most probable mechanism behind the coherent emission is the population inversion in the B^2^Σ_u_^+^ and X^2^Σ_g_^+^ states of N_2_^+^, and that nonlinear processes such as four wave mixing and stimulated Raman scattering in the intense laser fields are not involved^14^. Recent studies^6^,^12^ showed that the population inversion occurs on an ultrafast timescale comparable to the pump laser pulse (40–200 fs).

However, the underlying mechanism of building up the population inversion has not been convincingly explained since the first observation of the air lasing with N_2_^+^ as the gain medium^2^,^6^. This is because when a nitrogen molecule is exposed to an intense laser field, the ejection of an outer valence electron in the highest occupied molecular orbital (HOMO) of N_2_ leaves the ion N_2_^+^ in the ground X^2^Σ_g_^+^ state, whereas the ejection of an inner-valence electron in the HOMO-2 leads preferentially to the formation of N_2_^+^ in the excited B^2^Σ_u_^+^ state^15^. Since the ionization potential (*I*_p_=18.7 eV)^16^ for the removal of the HOMO-2 electron is much larger than that (*I*_p_=15.6 eV)^16^ for the removal of the HOMO electron of N_2_, it was theoretically predicted that the resultant yield of N_2_^+^ in the ground X^2^Σ_g_^+^ state in an intense laser field is much larger than that of N_2_^+^ in the excited B^2^Σ_u_^+^ state^17^, and thus, the population inversion of N_2_^+^ would not occur in intense laser fields. Therefore, previous studies mainly focused on the generation of N_2_^+^ lasing and experimental verification of population inversion^2^,^6^,^9^,^10^,^11^,^12^,^13^,^14^, and only some conjectures have been proposed for the population inversion mechanism, for example, different electron-ion recombination rates of ions in the B^2^Σ_u_^+^ state and the X^2^Σ_g_^+^ state^2^, and post-ionization Raman-like excitation^12^.

In the present study, we demonstrate the generation of the air lasing of N_2_^+^ at 391 nm by the irradiation of intense few-cycle 800 nm pulses into air. The formation of self-generated white-light seed amplification in the filamentation of a few-cycle laser pulse implies that it takes only 4–5 fs in achieving the population inversion. We consider various possible mechanisms that may contribute to the population inversion of N_2_^+^ and show, based on the results of numerical calculations, that the population inversion can be ascribed to the post-ionization optical coupling in the three-level system formed by the electronic ground state X^2^Σ_g_^+^ and the electronically excited states A^2^Π_u_ and B^2^Σ_u_^+^ in N_2_^+^. After ionization of N_2_ to produce N_2_^+^, the field-induced X^2^Σ_g_^+^–A^2^Π_u_ and X^2^Σ_g_^+^–B^2^Σ_u_^+^ couplings in the later part of the laser pulse can generate population inversion in the X^2^Σ_g_^+^(*ν*=0)–B^2^Σ_u_^+^(*ν*=0) transition. A key factor in the achievement of the population inversion is the presence of the A^2^Π_u_ state, which effectively acts as a trap for population transferred from the X^2^Σ_g_^+^ state through its lower dipole coupling strength.

## Results

### Experiment

**Figure 1:**
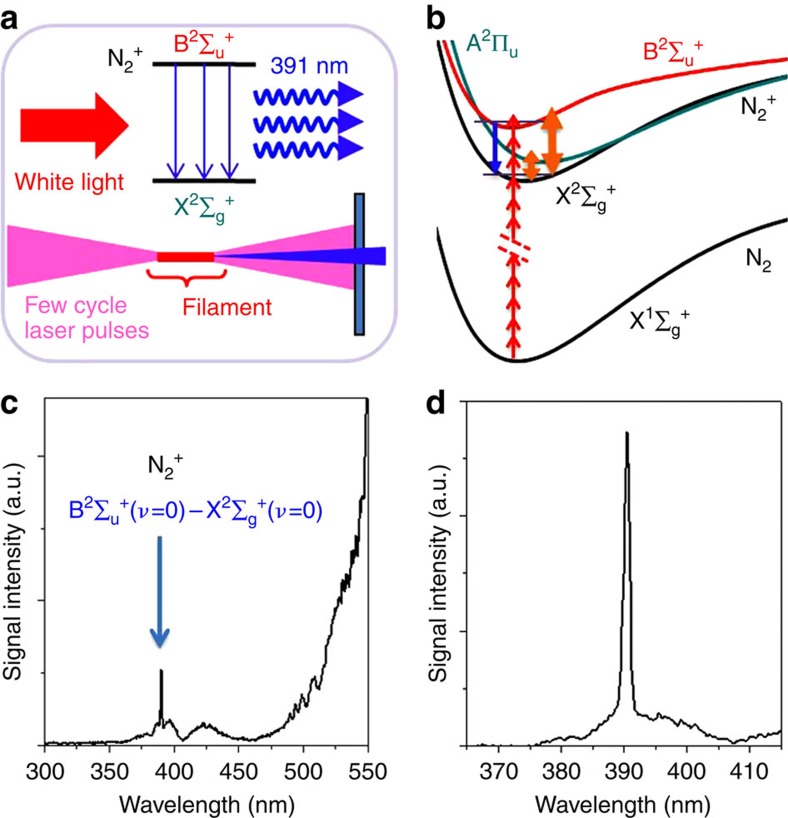
Air lasing concept and lasing emission. Schematics (**a**) of the lasing actions induced by the few-cycle laser pulse and the excitation scheme (**b**) of the population inversion with the potential energy surfaces^20^,^21^,^22^ of N_2_^+^, in which the upward red arrows denote the 800 nm laser photon, the downward blue arrow the lasing emission at 391 nm, and the double-headed arrows the dipole coupling induced by the laser field. (**c**) Forward spectrum of air lasing recorded together with the white light. (**d**) Spectrum of air lasing recorded forwardly with a band-pass filter.

We employ few-cycle laser pulses to produce the 391 nm lasing in ambient air (for experimental details, see Methods). The lasing actions and the excitation scheme of the population inversion of N_2_^+^ are schematically shown in Fig. 1a. The few-cycle pump pulse has two roles, that is, the ionization of the nitrogen molecules and the establishment of the population inversion of N_2_^+^. When the few-cycle pulse is focused in air, ionization of nitrogen molecules occurs with the laser intensity clamped inside the filament which is produced as a result of a dynamical interplay between Kerr self-focusing and defocusing of self-generated air plasma^18^. Meanwhile, the refractive index of air in the filament of the intense few-cycle laser pulse is modified, leading to the generation of new frequencies as a result of self-phase modulation^19^. It is found, as shown in Fig. 1b, that when the spectrum of the few-cycle pulse in the blue side becomes broad enough to cover the B^2^Σ_u_^+^(*ν*=0)→X^2^Σ_g_^+^(*ν*=0) transition of N_2_^+^, the narrow-band emission at 391 nm appears in the recorded forward laser spectrum. It can be seen from Fig. 1b that the intensity of the 391 nm band is about five times larger than that of the white light laser signal in the spectral range of 350–400 nm and no other fluorescence such as the strongest nitrogen signal at 337 nm can be observed. Our result is similar to the air lasing actions observed with long 40–200 fs pulses as the pump^6^,^9^, showing that the population inversion is achieved by the few-cycle laser pulse. Figure 1c shows the clean lasing spectrum with the white light filtered out by a band-pass filter, which is placed just before the fibre spectrometer.

### Theory

In order to simulate the population distribution of N_2_^+^ ions exposed to an intense laser pulse, we numerically solve the time-dependent Schrödinger equation for the nuclear wave function of N_2_^+^ (for details of the calculation, see Methods). We assume that at some instant during the laser pulse, an N_2_^+^ molecular ion is created by the tunnelling ionization of N_2_. After the ionization event, the N_2_^+^ molecular ion is still in the laser pulse, and therefore the field-induced coupling among the electronic states of N_2_^+^ changes the final populations of the excited states.

Our model includes non-perturbatively the dipole transitions (including resonant and Raman-type transitions) among all vibrational states on the three electronic states X^2^Σ_g_^+^, A^2^Π_u_ and B^2^Σ_u_^+^. The ground X^2^Σ_g_^+^ state is coupled to the excited A^2^Π_u_ state by the component of the laser field perpendicular to the molecular axis, while the ground state and the B^2^Σ_u_^+^ state couple via the parallel component. At the equilibrium internuclear distance *r*_e_≈1.12 Å of the X^2^Σ_g_^+^ state, the numerical values of the dipole couplings are^20^,^21^ 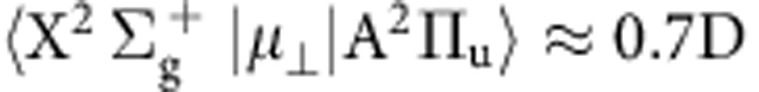 and 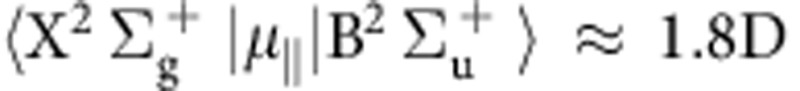. Due to the different symmetry, there is no direct coupling between the B^2^Σ_u_^+^ state and the A^2^Π_u_ state. Rotation of the molecule during the interaction with the laser field is neglected, since the rotational period is of N_2_ is about 8 ps (ref. 22).

**Figure 2:**
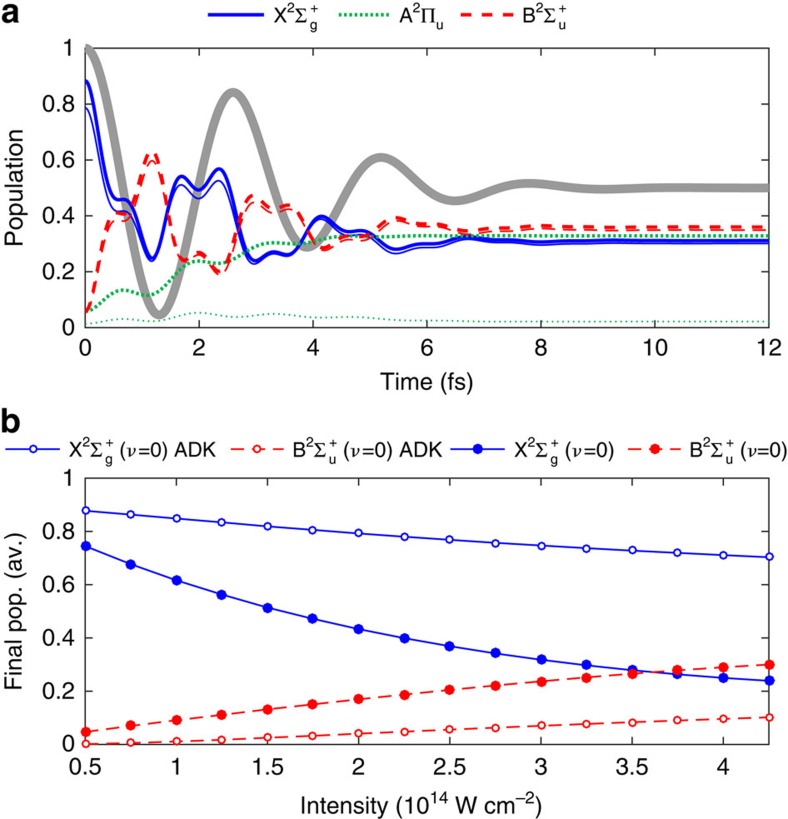
Population inversion of N_2_^+^ in a few-cycle laser pulse. (**a**) Time-dependent populations in the three electronic states, at a field intensity of 2 × 10^14^ W cm^−2^, and alignment angle *θ*=45°. Thick lines show the total population, while the thin lines show the population in the vibrational ground state. The laser field (not to scale) is indicated in the background as a fat, grey line. (**b**) Final populations averaged over alignment angle and ionization time, in the vibrational ground state of the X^2^Σ_g_^+^ state (solid lines), and of the B^2^Σ_u_^+^ state (broken lines). Closed symbols are the result of tunnelling ionization and post-ionization coupling, and open symbols show the result when only tunnelling ionization is included.

In Fig. 2a, we show an example of the time-dependent populations of the three electronic states, at the laser intensity of 2 × 10^14^ W cm^−2^, and where the angle between the laser polarization vector and the molecular axis is *θ*=45°. At this value of *θ* the component of the laser field parallel to the molecular axis equals the perpendicular component, which exemplifies the situation when the X^2^Σ_g_^+^ state is coupled both to the A^2^Π_u_ state and to the B^2^Σ_u_^+^ state, and leads to a particularly efficient creation of population inversion. We have assumed that tunnelling ionization from N_2_ to N_2_^+^ occurs at *t*=0 (at the peak of the laser pulse), and that the strong field-induced dynamics starts immediately after the ionization. The initial populations are taken to be proportional to the Ammosov–Delone–Krainov tunnelling rate of ionization^17^,^23^ to the respective electronic state. For the nuclear wave function, we adopted the Franck–Condon approximation and assumed the initial nuclear wave function to be identical to that of the vibrational ground state of N_2_. To make comparison with experiment, we average the final populations over the ionization time and over the alignment angle *θ* (assuming a randomly aligned molecular ensemble). The result of such an averaging is shown in Fig. 2b. In this figure, we also show a curve where we include only direct tunnelling ionization without post-ionization coupling.

**Figure 3:**
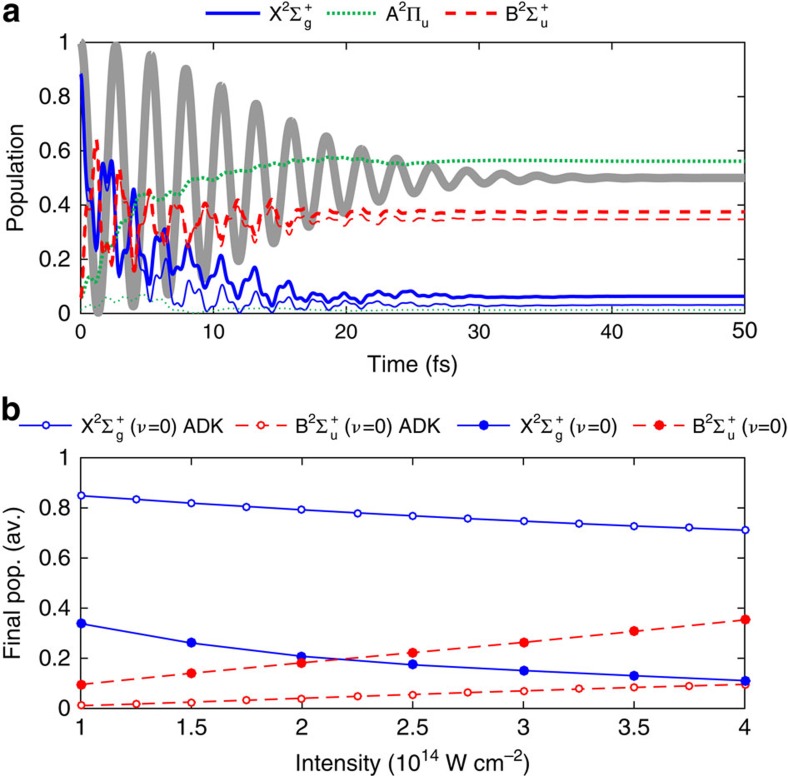
Population inversion of N_2_^+^ in a 20-fs laser pulse. (**a**) Time-dependent populations of the three electronic states in a laser pulse with a FWHM of 20 fs (other laser parameters as in Fig. 2), assuming ionization at *t*=0. Thick lines represent the total population in all vibrational states, and thin lines correspond to the populations in the vibrational ground states. The laser field (not to scale) is plotted in the background. (**b**) Final populations in the vibrational ground state of the X^2^Σ_g_^+^ state (solid lines), and of the B^2^Σ_u_^+^ state (broken lines). We compare the case when only tunnelling ionization is included (open symbols) and when tunnelling ionization and post-ionization dynamics is included (solid symbols).

We have also calculated the post-ionization coupled-state dynamics for a longer laser pulse. In Fig. 3a, we show the result of a calculation employing a pulse with a full-width at half-maximum (FWHM) of 20 fs, at an intensity of 2 × 10^14^ W cm^−2^ and an alignment angle of *θ*=45°. Figure 3b shows the result of averaging over ionization time and alignment angle.

## Discussion

The laser intensity in the experiments is estimated to be 4.2 × 10^14^ W cm^−2^. On the basis of previous measurements by Mitryukovskiy *et al*.^24^, for a collimated or weakly converging laser beam, the clamped intensity is about 1.4 × 10^14^ W cm^−2^, and for a tightly focused beam with a 10 cm focal length, the laser intensity is increased by about three times^25^. However, it can be seen in Fig. 2b that direct tunnelling ionization is insufficient to generate population inversion at the laser intensities of 4.2 × 10^14^ W cm^−2^.

In the following, we consider several possible scenarios that may contribute to the establishment of the population inversion of N_2_^+^ in a laser-induced filament. We show that only the mechanism proposed in the current paper, strong field-induced post-ionization coupling of the electronic states, is able to yield population inversion on sub-ps timescale.

The first possibility is that after ionization, N_2_^+^ molecules in the electronic ground state are excited by colliding with free electrons in the surrounding plasma. This mechanism has been shown to be the dominant one for the population of the B^2^Σ_u_^+^ state in filaments created by circularly polarized laser pulses^9^,^10^,^24^. However, given that the cross-section *σ* for the reaction e^−^+ N_2_^+^(X^2^Σ_g_^+^, *ν*=0)→e^−^+ N_2_^+^(B^2^Σ_u_^+^, *ν*=0) is of the order of 10^−16^ cm^2^ (see ref. 26), and that the typical free electron kinetic energy is 
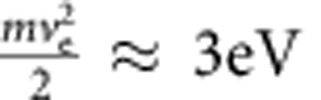
 (ref. 24), the resulting collision rate *R*_coll_=*ρ*_e_*v*_e_*σ*≈10^9^ s^−1^ (assuming an electron density *ρ*_e_=10^17^ cm^−3^ as measured in ref. 25). This means that the collision of the electrons with the surrounding nitrogen ions occurs on a nanosecond timescale, much slower than the duration of a femtosecond laser pulse. Therefore, we can exclude the contribution of this possibility to the ultrafast population inversion observed for the B^2^Σ_u_^+^ state and the X^2^Σ_g_^+^ state in N_2_^+^.

The second possibility which may contribute the population inversion is the recollision^27^ of the electron ejected from N_2_ with its parent N_2_^+^ ion, which could excite nitrogen ions from X^2^Σ_g_^+^ state to B^2^Σ_u_^+^(*ν*=0). The size of the recolliding electronic wave packet at recollision can be estimated to be^28^ 
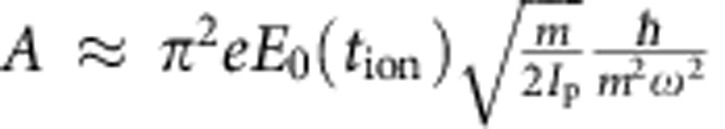
. In our case with an ionization potential of *I*_p_=15.6 eV and a peak intensity of 4 × 10^14^ W cm^−2^ we have *A* ≈ 8 × 10^−15^ cm^2^, much larger than the cross-section *σ*≈10^−16^ cm^2^ for the reaction e^−^+N_2_^+^(X^2^Σ_g_^+^,*ν*=0)→e^−^+N_2_^+^(B^2^Σ_u_^+^,*ν*=0)^26^. By calculating the recollision probability for an ejected electron as *σ*/*A*≈0.01, we estimate that the recollision has a negligible contribution to the population inversion of N_2_^+^.

The third possibility is the ‘post-ionization' optical couplings among the electronic excited states. As shown in Fig. 2a, the X^2^Σ_g_^+^ state and the B^2^Σ_u_^+^ state are strongly coupled, which results in a large population transfer occurring on a sub-cycle timescale. On the other hand, due to the smaller transition dipole matrix element, the population dynamics in the A^2^Π_u_ state is almost unidirectional: population is transferred to the A^2^Π_u_ state, but not back to the ground state again. Clearly, the strong field-induced coupling of the electronic states drastically increases the relative population of the B^2^Σ_u_^+^ state when compared with the results of the direct tunnel ionization, as shown by the open and closed symbols in Fig. 2b. After the laser pulse has passed, population inversion is achieved: the final population in the B^2^Σ_u_^+^(*ν*=0) state is larger than that in the X^2^Σ_g_^+^(*ν*=0) state. The calculated population inversion is achieved at a peak field intensity of 3.6 × 10^14^ W cm^−2^, which is smaller than the laser intensity of 4.2 × 10^14^ W cm^−2^ estimated from the current experimental condition, and therefore confirms the generation of the population inversion of N_2_^+^ within a few-cycle pulse. We point out that the average of the final population over the ionization time and over the alignment angle *θ* leads to an increase of the laser intensity to realize the population inversion as compared with that obtained in Fig. 2a. The reason is that at other alignment angles and ionization times than adopted in Fig. 2a, the population inversion is less efficient.

For the longer pulse case shown in Fig. 3, it can be seen from Fig. 3a that the final population in the B^2^Σ_u_^+^(*ν*=0) state is larger than that in the X^2^Σ_g_^+^(*ν*=0) state, and the difference of the populations between these two states is much larger when compared with that obtained in Fig. 2a. After the average of the final population over the ionization time and over the alignment angle *θ*, it can be seen from Fig. 3b that the population inversion can be achieved with a laser intensity of about 2.2 × 10^14^ W cm^−2^. This lowered intensity for the population inversion indicates the effects of the pulse duration on the population inversion of N_2_^+^, which may reflect an effectively stronger coupling between the excited state and the ground state of N_2_^+^ for a longer pulse. As described in ref. 29, the clamping intensity in air becomes higher when the pulse duration decreases from 200 to 42 fs. In the case of 5 or 20 fs pulses, the electronic responses of molecules are much faster, and the laser pulse may propagate with higher intensity, resulting in the higher clamping intensity than in the case of the longer pulse^29^. This is consistent with the threshold intensity of the population inversion shown in Figs 2b and 3b.

To examine the significance of the A^2^Π_u_ state, we carried out further simulations for both 5 and 20 fs laser pulses, assuming that there is no coupling between the X^2^Σ_g_^+^ state and A^2^Π_u_ states, that is, the population in the A^2^Π_u_ state is kept to be constant after the ionization; meanwhile, keeping all other parameters to be the same as those in the simulations of Figs 2 and 3. For both 5 and 20 fs laser pulses, we find after averaging over alignment angle and ionization time that the population inversion of N_2_^+^ between the X^2^Σ_g_^+^ state and B^2^Σ_u_^+^ state cannot be achieved in the intensity interval of 1 × 10^14^ W cm^−2^≤*I*≤4 × 10^14^ W cm^−2^. This result clearly shows the significance of the A^2^Π_u_ state, which can be regarded as a trap for the population transferred to the excited A^2^Π_u_ state of N_2_^+^.

We have also considered the contribution from the excited C^2^Σ_u_^+^ electronic state of N_2_^+^ in generating the population inversion of N_2_^+^. The C^2^Σ_u_^+^ state couples directly to the ground X^2^Σ_g_^+^ state via a parallel transition, but not to the A^2^Π_u_ and B^2^Σ_u_^+^ states. At the equilibrium internuclear distance of the ground X^2^Σ_g_^+^ state, the energy gap between the X^2^Σ_g_^+^ state and the C^2^Σ_u_^+^ state is^21^ 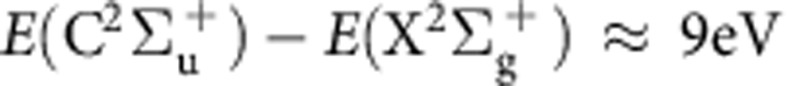, which is larger than 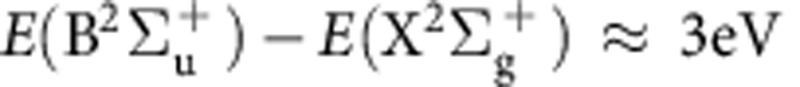 (ref. 21) and 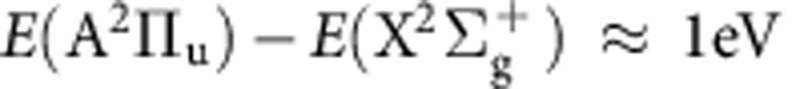 (ref. 20). The dipole matrix element is 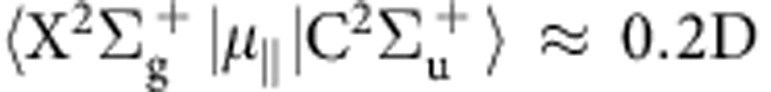. For the same parameters as used in Figs 2 and 3, we solved the Schrödinger equation including the four states X^2^Σ_g_^+^, A^2^Π_u_, B^2^Σ_u_^+^ and C^2^Σ_u_^+^, but no significant difference is observed when compared with those shown in Figs 2 and 3. The negligible contribution of the C^2^Σ_u_^+^ state can be ascribed to the large energetic gap between the X^2^Σ_g_^+^ and C^2^Σ_u_^+^ states, leading to a negligible coupling of the ground X^2^Σ_g_^+^ state with the excited C^2^Σ_u_^+^ state at the intensity of a few 10^14^ W cm^−2^.

In summary, we have demonstrated that the post-ionization strong field-induced dynamics of the three-level system formed by the X^2^Σ_g_^+^, B^2^Σ_u_^+^ and A^2^Π_u_ states in N_2_^+^ plays a key role in generation of the population inversion of N_2_^+^. The excited A^2^Π_u_ state is essential in the coupling, which serves as a ‘population reservoir' in the process of transferring the population into the B^2^Σ_u_^+^ state and depleting the population from the ground state. The proposed mechanism of post-ionization coupling can explain the formation of population inversion in N_2_^+^ irradiated with long, ∼100 fs laser pulses as employed in previous experiments^6^,^11^,^12^,^13^,^14^, but also implies that population inversion can be achieved using ultrashort, few-cycle pulses. We confirm this statement by observing that lasing in air can be produced even for a pulse as short as 4–5 fs.

## Methods

### Experimental details

We performed experiments with linearly polarized few-cycle laser pulses, which were produced from a femtosecond Ti:sapphire laser system (5 kHz, 0.6 mJ, 800 nm and 30 fs). Output pulses from the Ti:sapphire laser system were focused into a hollow-core fibre (inner diameter: 330 μm, length: 1.5 m) filled with argon gas at the pressure of 470 mbar to broaden the bandwidth of the pulse. After compressing the pulse by a set of chirped mirrors (PC70, Ultrafast Innovations) and two wedges, few-cycle pulses were generated (∼6 fs, 5 kHz and 0.2 mJ)^30^. The pulse duration of the few-cycle pulse was measured by an autocorrelator (FEMTOMETER). The generated few-cycle pulse was directly focused by a parabolic mirror (*f*=10 cm) in air to ionize the air molecules. A single filament of about 3 mm was produced. The forward light after the focus was collected by a lens (*f*=15 cm) and then focused into a fibre spectrometer (Ocean optics USB 4000-UV-VIS) in a 2*f*–2*f* manner.

### Numerical calculation

The population dynamics in the N_2_^+^ molecular ion is calculated by solving the time-dependent Schrödinger equation


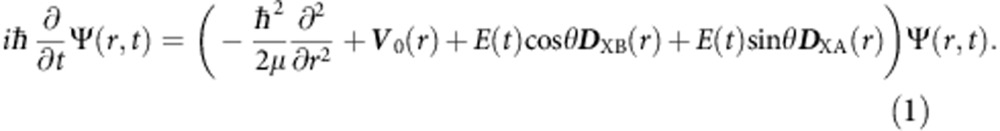


In Equation (1), 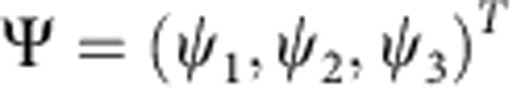 is a column vector containing the nuclear wave functions at the respective electronic surface (we have used the labelling 1 for the X^2^Σ_g_^+^ state, 2 for the A^2^Π_u_ state, and 3 for the B^2^Σ_u_^+^ state), *r* is used to denote the internuclear distance, *μ* is the reduced mass of N_2_^+^, *θ* is the angle between the molecular axis and the laser polarization direction, and *E*(*t*)=*E*_0_(*t*)cos(*ωt*) is the laser field. The potential ***V***_0_ is a 3 × 3 diagonal matrix with matrix elements (***V***_0_)_*ii*_=*v*_*i*_(*r*). The dipole transition matrices ***D***_XB_ and ***D***_XA_ are 3 × 3 matrices with the only non-vanishing matrix elements being (***D***_XB_)_13_=(***D***_XB_)_31_=*d*_13_(*r*), and (***D***_XA_)_12_=(***D***_XA_)_21_=*d*_12_(*r*). The potential energy curves *v*_1,2,3_(*r*) and the transition dipole moments *d*_12,13_(*r*) are taken from refs 20, 21. The dipole moment *d*_12_(*r*) changes smoothly from *d*_12_(*r*=1 Å)≈0.7D to *d*_12_(*r*=2 Å)≈0.1D, while *d*_13_(*r*) varies monotonically from *d*_12_(*r*=1 Å)≈2.0D to *d*_12_(*r*=2 Å)≈−1.0D.

To solve Equation (1) numerically, the wave functions are discretized on a grid with mesh width Δ*r*=0.0053 Å. We use a 3-point finite difference approximation for the kinetic energy operator. The time propagation is accomplished with the split-operator technique, using the Crank–Nicolson algorithm for the time-independent operators, and the Lanczos algorithm for the time-dependent ones, with a time step length of *δt*=12 as.

For all calculations, we employ a Gaussian envelope function *E*_0_(*t*) with an intensity FWHM of 5 fs, an angular frequency *ω*=2.4 rad s^−1^ (corresponding to a wavelength of 800 nm), and a CEP *φ*=0. The initial nuclear wave function is taken to be 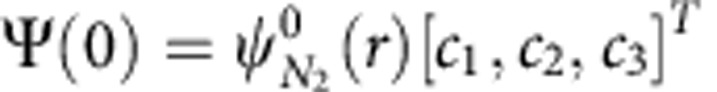, where 
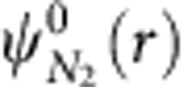
 is the vibrational ground state of the electronic ground state potential energy curve of the neutral N_2_ molecule^22^ (The Franck–Condon approximation), and *c*_1,2,3_ are constants specifying the relative populations of the electronic states.

The total population *P*_*i*_(*t*) in state *i* is defined as 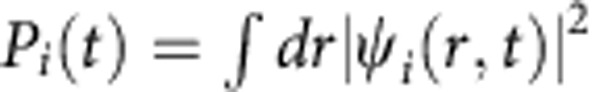, and satisfies 
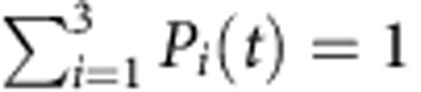
 at all times. In a similar way, the population 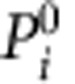 in the vibrational ground state of the electronic state *i* is calculated as 
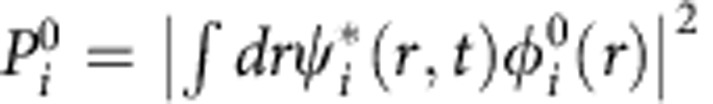
, where 
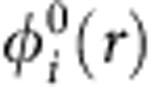
 is the ground state wave function of the potential energy curve *i*.

**Figure 4:**
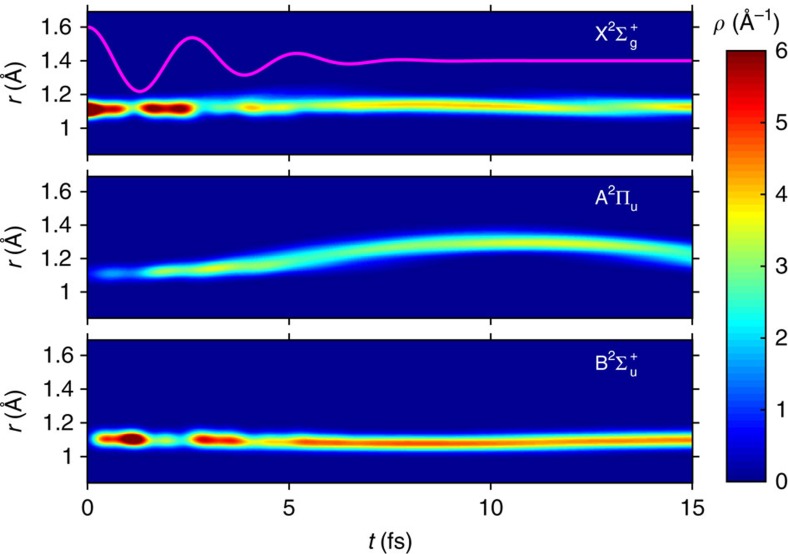
Time-dependent nuclear density 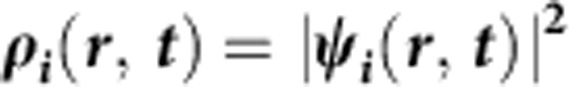. Nuclear densities in the X^2^Σ_g_^+^ state (top), the A^2^Π_u_ state (middle) and the B^2^Σ_u_^+^ state (bottom). The laser parameters used are the same as in Fig. 2a. In the top panel (showing the X^2^Σ_g_^+^ state), the laser field (on an arbitrary scale) is indicated as a solid line.

An example of the nuclear density 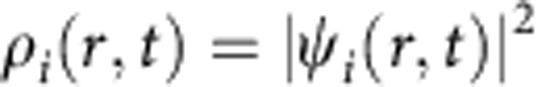 in each electronic state for the same laser parameters as used in Fig. 2a (*I*_0_=2 × 10^14^ W cm^−2^, *θ*=45°, FWHM=5 fs) is shown in Fig. 4. The strong coupling between the X^2^Σ_g_^+^ and the B^2^Σ_u_^+^ states is clearly visible. Also interesting is that since the equilibrium internuclear distance for the A^2^Π_u_ state is larger than that of the X^2^Σ_g_^+^ state (see the potential energy curves in Fig. 1a), vibrational motion is induced following the population transfer to the A^2^Π_u_ state. On the other hand, the nuclear wave functions in the X^2^Σ_g_^+^ state and the B^2^Σ_u_^+^ state have a large overlap with the respective vibrational ground state.

The averaging over the alignment angle *θ* and over ionization time *t*_0_ is performed in the following way. We define 
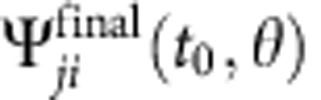
 as the final nuclear wave function in state *i*, calculated from an initial wave function given by 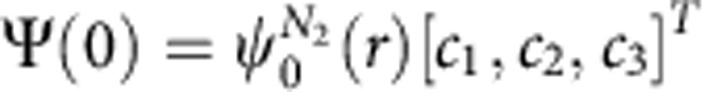, *c*_*j*_=1, *c*_*k*≠*j*_=0 (corresponding to an initial state with population only in state *j*), and at alignment angle *θ*. The incoherently averaged population 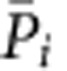 is then given by


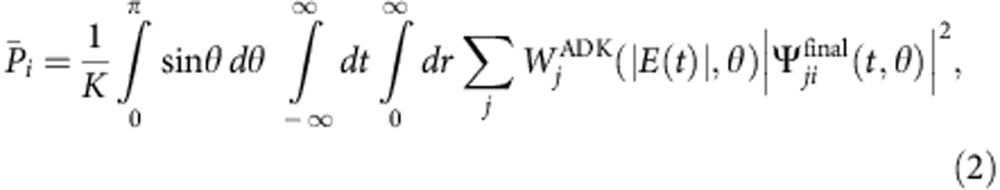


where 
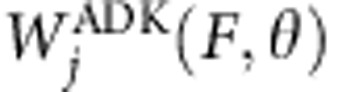
 is the Ammosov–Delone–Krainov ionization rate^17^ for tunnelling ionization to N_2_^+^ in electronic state *j*, at field strength *F* and alignment angle *θ*. *K* is a normalization constant to assure 
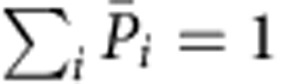
. In the case of the longer pulse shown in Fig. 3b, the integral over *t* in Equation (2) is approximated by a sum over the time instants of the laser field peaks. The averaged final populations assuming only tunnelling ionization (shown with open symbols in Fig. 2b) are similarly calculated by integrating 
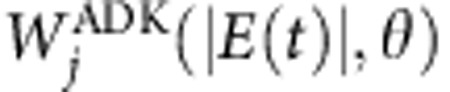
 over *t* and *θ*, and multiplying with the respective Franck–Condon factor.

## Additional information

**How to cite this article**: Xu, H. *et al*. Sub-10-fs population inversion in N_2_^+^ in air lasing through multiple state coupling. *Nat. Commun.* 6:8347 doi: 10.1038/ncomms9347 (2015).
